# Program evaluation of trauma-informed yoga for vulnerable populations

**DOI:** 10.1016/j.evalprogplan.2021.101946

**Published:** 2021-04-20

**Authors:** Deanne C. Tibbitts, Sue A. Aicher, Judith Sugg, Kimberlee Handloser, Liz Eisman, Lauren D. Booth, Ryan D. Bradley

**Affiliations:** a Helfgott Research Institute, National University of Natural Medicine, 049 SW Porter Street, Portland, OR, 97201, USA; b Oregon Health & Science University, 3181 SW Sam Jackson Park Road, Portland, OR, 97239, USA; c Living Yoga (Board of Directors: SAA, JS, Staff: LE, LDB, Volunteer: KH), 5100 SW Macadam Ave., Suite 360, Portland, OR, 97239, USA

**Keywords:** Program evaluation, Yoga, Psychological trauma, Stress disorders, Post-traumatic, Prisoners, Substance-related disorders

## Abstract

**Background:**

Trauma is highly prevalent among vulnerable populations, including those who are incarcerated, in treatment for substance use, or seeking mental health services. Trauma-informed yoga seeks to create a safer yoga practice for individuals with a trauma history and may improve emotional and physical wellbeing. Thus, we conducted an evaluation of a trauma-informed yoga program to gain insight into participant experiences.

**Methods:**

Trauma-informed yoga classes were led by trained volunteers and held in three sectors that work with vulnerable populations: corrections and reentry, substance use treatment and recovery, and community and mental health. Data were collected via anonymous survey using a retrospective pre-post design. The survey instrument captured reasons for student participation and perceived effects of yoga on emotional and physical wellbeing.

**Results:**

Students were motivated to participate in yoga classes by expectations of physical, mental, and spiritual benefit. Students reported perceived improvements in emotional and physical wellbeing and greater use of self-regulation skills after starting yoga.

**Conclusion:**

Our findings suggest trauma-informed yoga is perceived as beneficial by vulnerable individuals, especially those in the correctional system or recovering from substance use. Our results support the value of offering trauma-informed yoga in institutionalized and community settings. Improvements in emotional and physical wellbeing warrant formal study.

## 1. Introduction

Trauma results from an event that is temporarily overwhelming and exceptionally distressing, leaving lasting psychological symptoms (Briere & Scott, 2013). Many types of life experiences can lead to trauma, including sexual assault, interpersonal violence, child abuse, military combat, and natural disasters (Solomon & Davidson, 1997). While exposure to traumatic events can be found among the general population (Felitti et al., 1998; Kessler et al., 1995), it is highly prevalent among vulnerable populations, including those in the criminal (Carlson & Shafer, 2010; Jäggi et al., 2016; Salina et al., 2017) and juvenile justice systems (Abram et al., 2004; Dierkhising et al., 2013; McNair et al., 2019), those in treatment for alcohol or substance use (Giordano et al., 2016; Sanford et al., 2014; Simpson & Miller, 2002; Stewart, 1996; Wu et al., 2010), and those seeking mental health services (Cusack et al., 2006; Goodman et al., 1997; Mauritz et al., 2013). Trauma has been shown to have broad impacts on both mental and physical health, including increasing the risk of alcoholism and substance use (Felitti et al., 1998; Merrick et al., 2019; Wade et al., 2016), depression (Felitti et al., 1998; Merrick et al., 2019; Norman et al., 2012; Wade et al., 2016), heart disease (Jakubowski et al., 2018; Wade et al., 2016), cancer (Kelly-Irving et al., 2013; Merrick et al., 2019), and early mortality (Brown et al., 2009; Chen et al., 2016). Low cost, scalable approaches to treating trauma could therefore have a major impact on public health.

Yoga may be a potential treatment for addressing the effects of trauma. Systematic reviews report yoga shows promise as a helpful, low-risk intervention for trauma and related mental health concerns (Macy et al., 2018; Nguyen-Feng et al., 2019), though the overall evidence base lacks rigor. Small randomized controlled trials have shown that yoga can improve post-traumatic stress disorder (PTSD) symptomology (Carter et al., 2013; Jindani et al., 2015; Quinoñes et al., 2015; Seppälä et al., 2014; van der Kolk et al., 2014), anxiety (Jindani et al., 2015; Seppälä et al., 2014; Stoller et al., 2012), and insomnia (Jindani et al., 2015), and may improve depression (Nguyen-Feng et al., 2019), in people with trauma. A variety of mechanisms have been proposed to explain how yoga might improve trauma-related symptoms. Proposed mechanisms include both psychological and physiological effects, such as increased body awareness (Justice et al., 2018), improved emotion regulation (Dick et al., 2014), reduced inflammation, and improved regulation of both the hypothalamic-pituitary-adrenal axis and the autonomic nervous system (Kelly et al., 2018). However, very little research has been performed evaluating the effects of yoga in vulnerable populations in real-world settings.

One real-world setting in which yoga has been evaluated is in correctional facilities. Both trauma exposure and PTSD are associated with an increased likelihood of being incarcerated (Jäggi et al., 2016; Salina et al., 2017), and in addition to trauma-related mental health challenges, incarceration itself is an ongoing stressor. There is also a higher prevalence of substance use disorder among incarcerated individuals (National Center on Addiction and Substance Abuse, 2010). Studies of yoga in correctional facilities have shown yoga can improve mood, perceived stress, and psychological distress and reduce antisocial behavior among people experiencing incarceration (Bilderbeck et al., 2013; Kerekes et al., 2017). Previous studies of yoga for managing substance use disorders suggest yoga reduces substance-related cravings and increases self-reported abstinence (Kuppili et al., 2018). When taught in correctional and reentry settings, yoga and meditation may improve stress and reduce post-release substance use (Auty et al., 2017; Wimberly et al., 2018). Overall, yoga programs show promise for improving quality of life for people experiencing incarceration and for improving behaviors that may contribute to recidivism (Derlic, 2020; Muirhead & Fortune, 2016). However, the impact of larger scale yoga programs, outside of formal clinical trials, remains unknown.

Given the prevalence of trauma, an increasing number of service systems are taking a “trauma-informed” approach when working with clients. A trauma-informed approach seeks to create a safer environment for service delivery and to prevent re-traumatization (Substance Abuse and Mental Health Services Administration, 2014, pp. 9–11). In the context of yoga, a trauma-informed approach results in modifications to the typical yoga class environment and mode of instruction (Emerson et al., 2009; Justice et al., 2018). For example, a trauma-informed yoga class emphasizes safety, choice, and bodily autonomy for its students by using invitational language, providing options for each pose, and refraining from physical assists (Emerson et al., 2009). Ideally, trauma-informed yoga will create a safer environment in which students with a trauma history are supported in exercising choice and developing a friendly, non-antagonistic relationship with their bodies. A trauma-informed approach to yoga may be particularly important when working with vulnerable populations (Horton, 2017, p. 51; Murphy et al., 2019), as these individuals are more likely to have a history of trauma.

Here we describe results of an evaluation of a trauma-informed yoga program. Trauma-informed yoga classes were held in three sectors that work with vulnerable populations: corrections and reentry, substance use treatment and recovery, and community and mental health. The aims of the evaluation process were 1) to learn who is participating in the trauma-informed yoga program, 2) to assess the motivating factors for participating in trauma-informed yoga, and 3) to capture perceived changes in emotional and physical wellbeing from participating in trauma-informed yoga. We report the results of our program evaluation by sector to illustrate similarities and differences in program outcomes between the populations engaged.

## 2. Methods

### 2.1. Program characteristics

Each trauma-informed yoga class is led by a volunteer class facilitator who has completed a training program with Living Yoga. Volunteers are yoga practitioners themselves but are not necessarily professional yoga teachers. Volunteers receive intensive training, continuing education, and support in delivering trauma-informed yoga classes. The initial 17-hour training focuses on the physical and cognitive effects of trauma, how to structure a trauma-informed yoga class, and how to teach in an institutionalized or population-specific program setting. Following this initial training, new volunteers follow a stepwise process before leading their own trauma-informed yoga class. First, volunteers will observe trauma-informed yoga classes for two to three months at the partner site where they will be assigned, followed by co-teaching with an experienced volunteer at the same site. After one to three months of co-teaching and observation, new volunteers will be allowed to lead a class when they feel ready, and they will be observed by an established teacher or member of the Living Yoga training team for feedback and guidance. In addition to in-person support during yoga classes, volunteers receive additional support through semi-annual meetings with a cohort of volunteers who serve at similar facilities or specific sites. These cohort meetings provide updated information on best practices for teaching trauma-informed yoga and serve as an opportunity to share and gain wisdom from the teaching cohort. Examples of discussion topics include creative approaches to overcoming obstacles while teaching and resiliency tools for volunteers working in challenging settings (e.g. correctional facilities). Continuing education is offered to volunteers 6–8 times per year and is taught by Living Yoga staff and outside collaborators. Examples of continuing education topics include strategies for teaching specific populations (e.g. teens), building trauma resiliency, the intersection of mindfulness practices and social justice, and teaching to all body sizes.

Trauma-informed yoga classes are offered weekly at each partner site. Classes are held in multipurpose spaces, such as a cafeteria or classroom, rather than in a dedicated space for yoga. At some sites, spaces may be in a high traffic area and lack privacy. Spaces are not typically large enough to accommodate all interested students, therefore the class size is usually capped at 10–20 students depending on the facility and available space. Student access to props is limited to yoga mats, chairs, and blocks at most sites.

Classes are approximately one hour in duration and focus on calming the nervous system. Volunteers are trained to accommodate the students in the class within the context of available resources in the space. For example, a volunteer may use chairs, walls, blocks or other resources to make the yoga class accessible to each student. Accommodation is part of creating a trauma-sensitive environment, as meeting students where they are supports them in choosing their level of participation. No specific poses (i.e. asana) are required. Simple pose sequencing includes slow transitions, opportunities to notice sensations, and time for relaxation. Coordinated breath and movement sequences are commonly used. Volunteers frequently demonstrate poses, use verbal guidance of body movements rather than Sanskrit names of poses, and employ repetition of both verbal guidance and physical poses. Students are given several options for each pose with the stated understanding that they do not have to do any pose they do not feel comfortable doing. Students are asked to notice their breath even if they are not performing the poses. Physically challenging poses such as headstands and hand-stands are not included in classes in order to avoid injury and prevent a competitive atmosphere.

Key elements that establish a yoga class as trauma-informed include choice regarding level of participation, body awareness, and a relatively safe class environment. To promote choice, volunteers use invitational language and encourage students to modify or opt out of poses as needed. To promote body awareness, students may be asked to notice their feet or hands on the ground or to notice their breath. To promote a safe class environment, volunteers recognize and normalize a range of experiences, provide predictability, and promote self-ownership of body and body parts. Volunteers also promote safety by being attentive to how the room is organized, for example, by not placing students with their backs to the door. As these examples illustrate, teaching trauma-informed yoga requires volunteers to shift their priorities from teaching postures to fostering a sense of safety and grounding in the class – an approach that takes skill, training, and awareness on the part of the volunteer.

### 2.2. Program delivery setting

The trauma-informed yoga program is coordinated by Living Yoga, a 501(c)(3) non-profit organization. Living Yoga offers trauma-informed yoga classes at 23 partner sites in the Portland, Oregon metropolitan area. Collectively, these partner sites span adults and youth in both institutionalized and community settings. Partner sites can be divided into the following sectors: 1) corrections and reentry, 2) substance use treatment and recovery, and 3) community and mental health. The corrections and reentry sector includes minimum and medium security prisons, juvenile detention centers, and reentry programs. The substance use treatment and recovery sector includes residential treatment programs for adults and youth, community clinics, and recovery support programs. The community and mental health sector includes residential and community-based Child and Family Services centers and community clinics.

### 2.3. Program participation

Trauma-informed yoga classes are held at each partner site, and all individuals engaging with that site are eligible to attend classes. For instance, in institutionalized settings, all individuals in residence at a site may participate. In community settings, classes are held in public spaces, but classes are advertised to individuals engaging with that partner site. At most sites, class participation is voluntary. However, a subset of residential substance use treatment centers may mandate class participation. At sites where participation is voluntary, each site may choose to establish participation criteria. For example, in the corrections sector, participation may be limited to incarcerated individuals who do not demonstrate a behavioral risk and who have received approval to participate from prison administrators. In some youth settings, staff from the partner site may supervise classes and remove students whose behavior is disruptive. At some sites, student interest exceeds capacity, and so students remain on a wait-list until there is space available.

### 2.4. Survey instrument

The survey instrument (Appendix A) was developed by Living Yoga staff and Board members, including a psychologist, a public health professional, and individuals with lived experiences in the domains of service for our partner sites. The goal was to create a simple, accessible tool to measure the impact of trauma-informed yoga classes in domains including physical and emotional wellbeing and interoceptive awareness. Development of the instrument was informed by the framework of self-regulation proposed by trauma researchers Ford and Blaustein, in which they describe self-regulation as “the ability to (1) consciously focus attention; (2) be aware of the environment and one’s own physical and emotional body states; (3) draw on memory in order to learn from the past and adapt effectively in the present; and, (4) maintain or regain emotion states that provide a genuine sense of well-being and lead to further self-regulation” (Ford & Blaustein, 2013). For items querying physical and emotional wellbeing, each item is rated using a 5 point Likert scale, from “strongly disagree” to “strongly agree”. For items querying skill development and use, each action is rated using a 5-point Likert scale from “never” to “always”. The instrument also collects reasons for attending class, number of classes attended, and demographic information, including age, racial identity, gender identity, and sexual orientation.

### 2.5. Program evaluation

Data were collected from May 2018 to June 2018 as part of annual program evaluation activities conducted by Living Yoga. The survey used a retrospective pre-post approach (Pratt et al., 2000). Students completed the questionnaire at the end of yoga class, reporting on how they felt both before and after class. Students who had been to multiple yoga classes completed additional questions regarding how they felt both before and after starting yoga classes. Questionnaires were distributed only once at each partner site, to avoid individual students completing more than one evaluation. All students attending class on the date of evaluation were offered the opportunity to complete the program evaluation questionnaire. Questionnaires were completed anonymously. Students were not required to complete the questionnaire or to answer any questions they did not feel comfortable answering. Questionnaires were distributed by either the Living Yoga volunteer leading the class, a Living Yoga staff member, or a volunteer survey administrator at the end of class. Completed surveys were returned to the Living Yoga office for safe storage until results were compiled.

### 2.6. Data analysis

Univariate analysis was conducted on demographic characteristics. To assess motivating factors for attendance, responses were summed from all boxes checked. For items about physical and emotional well-being and development and use of skills, Likert scale responses were summed and percentages were calculated; results were then collapsed into dichotomous variables (agree/disagree). Responses are reported as percentage agreement with each statement (i.e., “agree” and “strongly agree”; “often” and “always”). For questions that were answered twice (retrospective pre-post), absolute differences were calculated by subtracting the percentage agreement before yoga from that after yoga. Partner sites were classified by sector, and analyses are reported for overall responses and responses by sector. Analyses were performed using STATA v15.1.

## 3. Results

### 3.1. Participant characteristics

The first aim of the evaluation was to learn who is participating in the trauma-informed yoga program. Of 152 students who completed the program evaluation, half (51 %; n = 77) attended trauma-informed yoga classes in the corrections and reentry sector, and the remainder were divided between the substance use treatment and recovery (21 %; n = 32) and community and mental health sectors (28 %; n = 43) (Table 1). The majority of survey respondents were adults (≥21 years; 73 %; n = 101) and female (59 %; n = 85). Students included representation from various communities of color (44 %; n = 64) and individuals who identified as gay, lesbian, bisexual, or pansexual (28 %; n = 37). The majority of survey respondents (82 %; n = 123) had attended more than one trauma-informed yoga class.

### 3.2. Missing data

Students were free to skip any question on the program evaluation, and we did not exclude any data based on incomplete questionnaires. The most frequently skipped demographic question asked about sexual orientation, with 13 % of respondents providing no answer (n = 20) (Table 1). For the remainder of the questionnaire, missing data did not exceed 4%.

### 3.3. Reasons for attending trauma-informed yoga classes

**Fig. 1.**
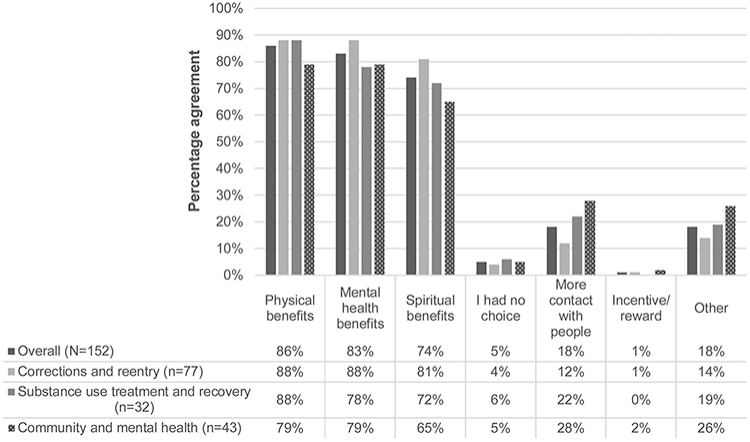
Reasons for attending trauma-informed yoga.

The second aim of the evaluation was to assess motivating factors for participating in trauma-informed yoga. When asked why they attend trauma-informed yoga, the majority of students reported being motivated by expectations of physical, mental, and spiritual benefits (Fig. 1). Fewer students (18 %; n = 28) indicated that they attend in order “to have more contact with people”. Free-response reasons for attending were reported by 18 % of respondents (n = 28); reasons for attending included “I’ve always wanted to learn yoga”, “help me relax”, “stretch out more sore ol’ muscles”, and “just the all around benefits”. Reasons for attending yoga were similar across sectors.

### 3.4. Perceived immediate benefits of the program

The third aim of the evaluation was to capture perceived changes in emotional and physical wellbeing from participating in trauma-informed yoga. We assessed changes in wellbeing over two time periods. First, all students were asked about perceived changes in well-being after a single yoga class. Second, if a student had attended more than one trauma-informed yoga class, they were also asked to evaluate perceived changes in wellbeing since starting yoga. As shown in Table 1, the number of classes attended by returning students ranged from two classes to more than 10.

When asked about perceived changes after a single yoga class, the proportion of students reporting awareness of physical sensations, such as breathing and muscle movement, increased compared to before class and the proportion of students reporting feeling pain or negative emotional states decreased (Table 2). For physical sensations, an additional 30 % (n = 46) and 23 % (n = 35) of students reported awareness of their breath and muscle movements, respectively, after class. For students reporting pain, the proportion dropped from 51 % (n = 76) before class to 18 % (n = 27) after class – an absolute difference of 33 %. For negative emotional states, only 6% (n = 9) of students reported feeling upset, anxious, or stressed after class. When looking at responses by sector, the proportion of agreement with each statement was similar, with two notable exceptions: fewer respondents from substance use treatment and recovery sites retrospectively reported feeling upset (10 % agreement vs mean of 25 %) and anxious or stressed (23 % agreement vs mean of 39 %) before yoga class.

### 3.5. Perceived longer-term benefits of the program

Students who had attended more than one trauma-informed yoga class (n = 123) were surveyed about the development and use of self-regulation skills that they apply in their daily lives (Table 3). When considering how often they used self-regulation skills before starting yoga classes, fewer than half the students indicated that they could deal with negative feelings, deal with stressful situations easily, or take healthy actions in response to how they feel. Since beginning yoga classes, use of self-regulation skills was uniformly higher, with an additional 18–36 % of students indicating use of these skills.

Looking at survey responses by sector, we found students in the corrections and reentry sector had the lowest reported use of self-regulation skills before starting yoga and had the largest increase in use after beginning yoga (Table 3). For example, before beginning yoga, 29 % of students in corrections and reentry programs (n = 20) indicated that “When I notice my feelings, I choose how to act in a healthy way”, which increased to 79 % (n = 54) after beginning yoga – an absolute difference of 50 %. Conversely, students in the substance use treatment and recovery sector had the highest reported use of self-regulation skills before starting yoga and showed the least amount of change in use after starting yoga.

**Fig. 2.**
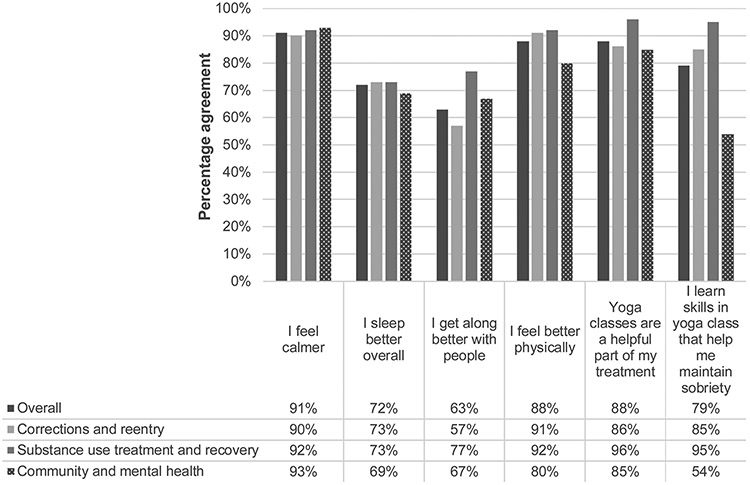
Perceived benefits of attending trauma-informed yoga. N = 123 for all statements, except treatment and sobriety (N = 109).

Students who had attended more than one class (n = 123) were also surveyed about perceived benefits of attending yoga classes (Fig. 2). Students endorsed wide-ranging benefits, including an increased sense of calm, better sleep, better social interactions, and better physical wellbeing. For students who are in recovery from substance use (n = 109), the majority of students indicated that yoga is a helpful part of their treatment (88 %; n = 96) and that they learn skills in yoga class that help them maintain sobriety (79 %; n = 84). Student responses were largely the same across sectors, with the exception of fewer students from community and mental health sites agreeing that they learn skills in yoga class that help maintain sobriety (54 % agreement vs mean of 79 %).

## 4. Discussion

The purpose of our program evaluation was 1) to learn who is participating in the trauma-informed yoga program, 2) to assess the motivating factors for participating in trauma-informed yoga, and 3) to capture perceived changes in emotional and physical wellbeing from participating in trauma-informed yoga. Our evaluation had broad representation from students participating in the program – the evaluation was completed by students at 23 partner sites across three sectors. The evaluation also captured a broad demographic of students, including individuals with a range of racial/ethnic, gender, and sexual orientation identities who are generally less represented in yoga classes. The majority of students completing the evaluation were repeat attendees, allowing us to capture both immediate and longer-term perceived benefits of trauma-informed yoga. We found that regardless of sector, students were motivated to participate by expectations of physical, mental, and spiritual benefit. Consistent with those expectations, we also found students reported perceived improvements in emotional and physical wellbeing and greater use of self-regulation skills after starting trauma-informed yoga classes compared to before.

For people with a history of trauma, trauma-informed yoga may be a vehicle for developing self-regulation, body awareness, and skills that aid in coping with stress. After a single yoga class, more students reported being aware of physical sensations, such as breathing and muscle movement, and fewer reported feeling negative emotional states, after class compared to before class. For students who had attended multiple yoga classes, more students reported noticing feelings, making healthier choices in response to feelings, and being able to deal with negative feelings and stressful situations after repeated yoga practice compared to before attending classes. Student reports of improved self-regulation support previous findings of trauma-informed yoga improving emotion regulation in individuals with a history of trauma (Dick et al., 2014). Our results also support previous findings that improved body awareness through the use of yoga is helpful for individuals experiencing PTSD and other forms of trauma (Justice et al., 2018; Price et al., 2017; van der Kolk et al., 2014). We found that students in the corrections and reentry setting reported the largest improvement in self-regulation skills after participating in yoga. These results are in line with other studies of yoga and meditative practices in a correctional setting, which demonstrated that these practices improve psychological wellbeing and behavioral functioning of people experiencing incarceration (Auty et al., 2017; Sfendla et al., 2018).

It has been suggested that the beneficial impact of yoga on mental health is greatest if yoga practice is part of a practitioner’s worldview, meaning they are immersed in yoga philosophy as well as physical asana (Gaiswinkler & Unterrainer, 2016). Our results suggest that yoga can also have substantial impact on students who are fairly new to the practice and who have limited access to yoga. Our students reported perceived improvements in sleep, mood, social interactions, and general physical wellbeing after attending multiple trauma-informed yoga classes, which is consistent with studies showing improvement in positive and negative affect (Jindani et al., 2015), anxiety (Jindani et al., 2015; Seppälä et al., 2014; Stoller et al., 2012), and insomnia (Jindani et al., 2015) after yoga in individuals with a history of trauma. Yoga has also been shown to improve various types of chronic pain (Tick et al., 2018). Half of our participants reported bodily pain prior to engaging in yoga class, consistent with the prevalence of chronic pain in the general population (Nahin et al., 2019). After a single class, students reporting pain fell to 18 %, showing that even novice students report pain reduction when being taught in a trauma-informed approach.

Our findings suggest trauma-informed yoga practice has broad benefits for institutionalized individuals. A future goal of our work is to determine if the positive impacts of yoga are sustained after individuals have left the institutional setting, which will require collaborative tracking of individual students and assessment of long-term outcomes. Potential outcomes include relapse prevention (Grow et al., 2015), recidivism, and self-reports of improvement in physical and mental health. Improvements in each of these areas would have significant positive impacts on individuals and their communities. While long-term impacts require future study, our current results show participants perceive positive effects from this program, which supports the use of trauma-informed yoga for individuals who are incarcerated and in substance use and mental health treatment facilities.

Our findings support the feasibility and scalability of delivering trauma-informed yoga in group classes taught by trained volunteers. Studies of yoga often involve classes delivered by yoga instructors with formal teacher training, are of limited duration, and are offered at high cost. In contrast, the program evaluated here is delivered by trained volunteers at low or no cost to the students. In addition, our training of volunteer class facilitators emphasizes trauma-informed best practices, accessibility, inclusivity, and authentic connection. As such, the sequences of poses offered in each class are not fixed but are adapted to the setting and the participants. Despite this variability, the beneficial impacts were seen widely across participating sectors. Our findings demonstrate that trained volunteers are capable of delivering trauma-informed yoga classes, suggesting trauma-informed yoga could be adapted to larger populations in a cost-effective manner.

## 5. Lessons learned

As Living Yoga’s trauma-informed program has been developed and refined over the past 20 years, anecdotal feedback has suggested that students derive a multitude of benefits from participating in trauma-informed yoga classes. Students have reported decreased stress and anxiety, improved emotional regulation, increased distress tolerance, improved self-confidence, improved impulse control, and increased compassion for self and others, as well as reduced pain, better sleep, and improved physical conditioning. Staff at partner sites have reported fewer discipline problems and better sleep in individuals who participated in yoga classes. We undertook our program evaluation to begin to quantify these reported benefits and to capture insights into possible mechanisms for the beneficial effects.

Given the vulnerable populations that participate in our trauma-informed yoga program, conducting a successful program evaluation required us to balance methodological choices against feasibility. Therefore, our program evaluation had some limitations. First, data were not collected using a validated instrument. We realized that using a battery of validated instruments to capture the wide range of potential physical, emotional, and mental health improvements would not be feasible in terms of time and resources. Instead, we chose to develop a simple evaluation tool that would: 1) capture a broad range of domains of potential impact to give us a sense of the perceived benefits of the program, 2) be accessible to a broad age range (i.e., both adults and youth) and across a wide range of settings, and 3) be completed quickly (i.e., less than 10 min), all of which were critical to the success of the evaluation process. However, because the instrument was not validated, the conclusions that we can draw about the impacts of trauma-informed yoga on specific physical, emotional, and mental health constructs are limited. We also did not explicitly assess trauma in our yoga students; however, studies from the criminal and juvenile justice system, alcohol and substance use treatment centers, and the mental healthcare system demonstrate trauma is highly prevalent among these populations (Abram et al., 2004; Carlson & Shafer, 2010; Cusack et al., 2006; Dierkhising et al., 2013; Giordano et al., 2016; Goodman et al., 1997; Jäggi et al., 2016; Mauritz et al., 2013; McNair et al., 2019; Salina et al., 2017; Sanford et al., 2014; Simpson & Miller, 2002; Stewart, 1996; Wu et al., 2010). Notably, the program was well-received by a majority of participants, irrespective of specific personal trauma history.

Second, we collected data using a retrospective pre-post design. This approach allowed us to maximize survey response by minimizing the burden on both students and program evaluation staff. Our design also compensated for response-shift bias, a phenomenon in which an individual’s understanding or interpretation of survey questions can change as a result of program participation, which threatens the validity of pre-test responses (Howard & Dailey, 1979). Indeed, we observed response-shift bias in an earlier version of our evaluation when we collected data prospectively; students reported that they had overestimated in their initial responses to the survey, leading to underestimation of effects of the yoga program. However, data from retrospective pre-post designs can be complicated by other types of bias, including social desirability and recall bias (Geldhof et al., 2018), leading to overestimation of effects. Results should therefore be interpreted with caution.

Third, we chose to capture data using a cross-sectional approach despite being interested in changes in physical, emotional, and mental health over time. We realized from the beginning of our development process that capturing longitudinal evaluation data would be challenging due to the drop-in/drop-out nature of some of the settings. For example, students from in-patient substance use treatment programs may only be in residence for 30 days. Long-term follow-up data from students who participate regularly would be best collected in settings where people are in one setting for a longer period of time (e.g., correctional facilities). However, longitudinal follow-up is also challenging when balanced against the privacy concerns of the individuals who participate. Capturing data at a single time point was also more feasible for funding and staffing reasons as well as for ensuring that survey responses were not duplicated.

Another limitation of this study is the self-selecting nature of most participants. Not everyone at each site is interested in yoga, so the students who filled out the survey are a self-selected subset of people at each site who 1) choose to participate in yoga classes and 2) continue to return (with the exception of first-time attendees). Results from the evaluation must be interpreted in this light; we do not know how people who may have stopped coming to class would respond to the survey.

Finally, we cannot report a survey response rate because survey participation was voluntary and anonymous. However, over several years of piloting our student survey, we learned that certain factors were important for maximizing survey participation. We found that it was important for the survey to be distributed by someone who could explain its purpose, answer any questions, and reinforce the voluntary and anonymous nature of the survey. Yoga students were less likely to complete the survey if they were not told the rationale for the survey, if no one was available to answer their questions, and/or if the anonymous nature of the survey was not emphasized. Anecdotally, if these conditions were met, most students who attended class chose to complete the survey.

## 6. Conclusions

The purpose of this program evaluation was to learn about motivating factors and perceived benefits to emotional and physical wellbeing from students participating in a trauma-informed yoga program. Our findings suggest trauma-informed yoga was perceived as beneficial for vulnerable individuals, especially those in the correctional system or recovering from substance use. Delivery of trauma-informed yoga in both institutionalized and community settings provides access to yoga for vulnerable populations who do not typically participated in yoga. Trauma-informed yoga classes were viewed favorably by students who participated, the majority of whom were repeat attendees. These results support the value of offering trauma-informed yoga in both institutionalized and community settings. Formal study is warranted to better define and quantify potential improvements in physical and emotional wellbeing and the potential of trauma-informed yoga to support recovery from substance use.

## Supplementary Material

1

## Figures and Tables

**Table 1:** Sociodemographic characteristics of survey respondents.

		By program evaluation setting		
	Overall (N = 152)	Corrections and reentry (n = 77)	Substance use treatment and recovery (n = 32)	Community and mental health (n = 43)
	n (%)	n (%)	n (%)	n (%)
**Age** (n = 139)				
Adult (≥ 21 years)	101 (73 %)	64 (86 %)	18 (67 %)	19 (50 %)
Youth (< 21 years)	38 (27 %)	10 (14 %)	9 (33 %)	19 (50 %)
**Gender** (n = 145)^a^				
Female	85 (59 %)	48 (64 %)	15 (50 %)	22 (55 %)
Male	53 (37 %)	26 (35 %)	14 (47 %)	13 (33 %)
Non-binary	4 (3 %)	1 (1 %)	0 (0 %)	3 (8 %)
Transgender	2 (1 %)	0 (0 %)	1 (3 %)	1 (3 %)
Other	1 (1 %)	0 (0 %)	0 (0 %)	1 (3 %)
**Race/ethnicity** (n = 144)^a^				
Native American, American Indian, or Alaskan Native	5 (4 %)	3 (4 %)	1 (3 %)	1 (3 %)
Asian or Asian American	4 (3 %)	1 (1 %)	2 (7 %)	1 (3 %)
Black or African American	4 (3 %)	3 (4 %)	1 (3 %)	0 (0 %)
Hispanic, Latino/a, or Spanish origin	14 (10 %)	7 (9 %)	2 (7 %)	5 (13 %)
Native Hawaiian or other Pacific Islander	1 (1 %)	1 (1 %)	0 (0 %)	0 (0 %)
White	80 (56 %)	43 (57 %)	19 (63 %)	18 (46 %)
More than one race/ethnicity	29 (20 %)	14 (19 %)	4 (13 %)	11 (28 %)
Other race, ethnicity, or origin	7 (5 %)	3 (4 %)	1 (3 %)	3 (8 %)
**Sexual orientation** (n = 132)				
Straight/Heterosexual	95 (72 %)	57 (79 %)	21 (81 %)	17 (50 %)
Gay or Lesbian/Homosexual	5 (4 %)	1 (1 %)	1 (4 %)	3 (9 %)
Bisexual	25 (19 %)	12 (17 %)	4 (15 %)	9 (26 %)
Pansexual	4 (3 %)	0 (0 %)	0 (0 %)	4 (12 %)
Unsure	3 (2 %)	2 (3 %)	0 (0 %)	1 (3 %)
**Total classes attended** (n = 150)				
One	27 (18 %)	9 (12 %)	8 (25 %)	10 (24 %)
Two to five	43 (29 %)	21 (27 %)	12 (38 %)	10 (24 %)
Six to ten	23 (15 %)	14 (18 %)	3 (9 %)	6 (15 %)
More than ten	57 (38 %)	33 (43 %)	9 (28 %)	15 (37 %)

a Responses do not sum to 100 % due to rounding.

**Table 2:** Perceived changes following a single trauma-informed yoga class.

	Agreed with statement n (%)						
	I felt my inhale and my exhale when I took a breath	I could feel my musdes working for me	I felt good about myself	I felt in control of my body	I felt pain in my body	I felt upset	I felt anxious or stressed
**Overall (N = 152)**							
Before class	93 (62 %)	101 (67 %)	95 (63 %)	97 (65 %)	76 (51 %)	37 (25 %)	57 (39 %)
After class	139 (92 %)	136 (90 %)	126 (83 %)	129 (85 %)	27 (18 %)	9 (6 %)	9 (6 %)
**Absolute difference**	30 %	23 %	20 %	20 %	−33%	−19%	−33%
**Corrections and reentry (n = 77)**							
Before class	41 (53 %)	53 (69 %)	51 (67 %)	45 (59 %)	39 (51 %)	18 (24 %)	30 (39 %)
After class	68 (88 %)	67 (87 %)	66 (87 %)	68 (88 %)	11 (14 %)	1 (1 %)	1 (1%)
**Absolute difference**	35 %	18 %	20 %	29 %	−37%	−23%	−38%
**Substance use treatment and recovery (n = 32)**							
Before class	23 (72 %)	21 (68 %)	21 (68 %)	25 (78 %)	16 (52 %)	3 (10 %)	7 (23 %)
After class	32 (100 %)	30 (94 %)	27 (84 %)	26 (81 %)	6 (19 %)	2 (6 %)	1 (3 %)
**Absolute difference**	28 %	26 %	16 %	3 %	−33 %	−4 %	−20 %
**Community and mental health (n = 43)**							
Before class	29 (69 %)	27 (63 %)	23 (53 %)	27 (64 %)	21 (51 %)	16 (40 %)	20 (50 %)
After class	39 (93 %)	39 (91 %)	33 (77 %)	35 (83 %)	10 (24 %)	6 (15 %)	7 (18 %)
**Absolute difference**	24 %	28 %	24 %	19 %	−27%	−25%	−32%

**Table 3:** Perceived changes in self-regulation skills since beginning trauma-informed yoga.

	Agreed with statement n (%)					
	I notice my feelings	When I notice my feelings, I choose how to act in a healthy way	I feel in control	I stretch or take a breath when I am uncomfortable	I deal with stressful situations easily	I can deal with negative feelings
**Overall (N = 123)**						
Before yoga	70 (58 %)	47 (39 %)	52 (43 %)	51 (43 %)	35 (29 %)	45 (38 %)
After yoga	94 (76 %)	91 (75 %)	91 (75 %)	94 (78 %)	69 (57 %)	76 (63 %)
**Absolute difference**	18 %	36 %	32 %	35 %	28 %	25 %
**Corrections and reentry (n = 68)**						
Before yoga	36 (53 %)	20 (29 %)	24 (35 %)	27 (40 %)	15 (22 %)	22 (32 %)
After yoga	53 (78 %)	54 (79 %)	54 (79 %)	53 (79 %)	42 (62 %)	47 (69 %)
**Absolute difference**	25 %	50 %	44 %	39 %	40 %	37 %
**Substance use treatment and recovery (n = 24)**						
Before yoga	15 (65 %)	14 (61 %)	14 (61 %)	12 (55 %)	11 (48 %)	14 (61 %)
After yoga	18 (75 %)	17 (71 %)	16 (67 %)	18 (75 %)	11 (46 %)	16 (67 %)
**Absolute difference**	10 %	10 %	6 %	20 %	−2 %	6 %
**Community and mental health (n = 31)**						
Before yoga	19 (63 %)	13 (42 %)	14 (45 %)	12 (40 %)	9 (30 %)	9 (31 %)
After yoga	23 (74 %)	20 (67 %)	21 (72 %)	23 (77 %)	16 (53 %)	13 (45 %)
**Absolute difference**	11 %	25 %	27 %	37 %	23 %	14 %
