# Supplementary material for: Program evaluation of trauma-informed yoga for vulnerable populations
Source: Eval Program Plann. Author manuscript; Available in PMC 2021 Oct 1. (PMC8325936; doi:10.1016/j.evalprogplan.2021.101946)
Supplement: 1 [file NIHMS1697474-supplement-1.pdf]

Living Yoga

Living Yoga Student Survey
Attended 2 or More Living Yoga Classes

We would like to learn more about you and your experience with Living Yoga. Your answers are kept confidential and will be used for program improvement and promotion. Participation is voluntary and you may skip any question you don't want to answer. We appreciate the time you spend completing this survey.

How many Living Yoga classes have you attended?

- ☐ This was my first Living Yoga class.
- ☐ 2-5 Living Yoga classes
- ☐ 6-10 Living Yoga classes
- ☐ More than 10 Living Yoga classes

Why did you come to Living Yoga class today? Check all that apply.

- ☐ For the physical benefits
- ☐ For the mental health benefits
- ☐ For the spiritual benefits (for ex., sense of connectedness, inner peace, feeling more centered)
- ☐ Because I had no choice
- ☐ To have more contact with people
- ☐ Because there was an incentive/reward
- ☐ Other ______________________________

Please mark the circle that best describes how much you disagree or agree with the following statements:

***Before the Living Yoga class today*. . .**

	Strongly disagree	Disagree	Neutral	Agree	Strongly agree
I felt my inhale and my exhale when I took a breath.	○	○	○	○	○
I could feel my muscles working for me.	○	○	○	○	○
I felt pain in my body.	○	○	○	○	○
I felt upset.	○	○	○	○	○
I felt anxious or stressed.	○	○	○	○	○
I felt good about myself.	○	○	○	○	○
I felt in control of my body.	○	○	○	○	○

***After the Living Yoga class today*. . .**

	Strongly disagree	Disagree	Neutral	Agree	Strongly agree
I am happy I came to the Living Yoga class today.	○	○	○	○	○
I would like to go to another Living Yoga class.	○	○	○	○	○
I feel my inhale and my exhale when I take a breath.	○	○	○	○	○
I can feel my muscles working for me.	○	○	○	○	○
I feel pain in my body.	○	○	○	○	○
I feel upset.	○	○	○	○	○
I feel anxious or stressed.	○	○	○	○	○
I feel good about myself.	○	○	○	○	○
I feel in control of my body.	○	○	○	○	○

Please mark the circle that most closely matches how often you did/do each of the following actions -

Before ever coming to your first Living Yoga class.

	Never	Rarely	Sometimes	Often	Always
I noticed my feelings.	○	○	○	○	○
When I noticed my feelings, I chose how to act in a healthy way.	○	○	○	○	○
I felt in control.	○	○	○	○	○
I stretched or took a breath when I was uncomfortable.	○	○	○	○	○
I dealt with stressful situations easily.	○	○	○	○	○
I could deal with negative feelings.	○	○	○	○	○

Since you started coming to Living Yoga classes.

	Never	Rarely	Sometimes	Often	Always
I notice my feelings.	○	○	○	○	○
When I notice my feelings, I choose how to act in a healthy way.	○	○	○	○	○
I feel in control.	○	○	○	○	○
I stretch or take a breath when I am uncomfortable.	○	○	○	○	○
I deal with stressful situations easily.	○	○	○	○	○
I can deal with negative feelings.	○	○	○	○	○

Please mark the circle that best describes how much you disagree or agree with the following statements

Since you started coming to Living Yoga classes.

	Strongly disagree	Disagree	Neutral	Agree	Strongly agree	Doesn't apply
I feel calmer.	○	○	○	○	○	
I sleep better overall.	○	○	○	○	○	
I get along better with people.	○	○	○	○	○	
I feel better physically.	○	○	○	○	○	
Living Yoga classes are a helpful part of my treatment.	○	○	○	○	○	N/A
I learn skills in Living Yoga class that help me maintain sobriety.	○	○	○	○	○	N/A

Is there anything else you would like to let us know about your experience with Living Yoga?

To be mindful and holistic in our services, the following information is helpful. All questions are optional and responses are kept confidential.

Age: _______

How do you identify your gender?

- ▯ Female
- ▯ Male
- ▯ Non-binary
- ▯ Transgender
- ▯ Other: __________

How do you identify your sexual orientation?

- ▯ Gay or Lesbian/Homosexual
- ▯ Straight/Heterosexual
- ▯ Bisexual
- ▯ I'm not sure
- ▯ I prefer not to say

I identify as a Person of Color.

- ▯ Yes
- ▯ No
- ▯ I'm not sure

How would you describe yourself? Check ***all that apply*** to your racial or ethnic identity.

- ▯ Native American or American Indian or Alaskan Native
- ▯ Asian or Asian American
- ▯ Black or African American
- ▯ Hispanic, Latino/a, or Spanish origin
- ▯ Middle Eastern or North African
- ▯ Native Hawaiian or Other Pacific Islander
- ▯ White
- ▯ Other race, ethnicity, or origin: ____________________

Office Use Only
Site: _____________________
Date: ____________________
